# Gambling Marketing Strategies and the Internet: What Do We Know? A Systematic Review

**DOI:** 10.3389/fpsyt.2021.583817

**Published:** 2021-02-26

**Authors:** Morgane Guillou-Landreat, Karine Gallopel-Morvan, Delphine Lever, Delphine Le Goff, Jean-Yves Le Reste

**Affiliations:** ^1^EA 7479 SPURBO, University Bretagne Occidentale, Brest, France; ^2^UMR SPHERE 1246, University Nantes/Tours, Nantes, France; ^3^HUGOPSY Network, Rennes, France; ^4^Addictive Disorders Center, Brest, France; ^5^EHESP, School of Public Health, CREM UMR CNRS 6211, Rennes, France

**Keywords:** gambling, marketing, online, betting, advertising

## Abstract

**Background:** The gambling industry has developed many types of gambling on Internet in recent years. Gambling is a social activity for a majority of the world population, but problem gambling (PG) can emerge. The trajectories of gamblers from initiation to PG development are influenced by many variables, including individual and environmental variables and also variables linked to the gambling characteristics. Marketing has been reported to influence gamblers' perceptions and behaviors, but this is not as clear for digital marketing. Digital gambling marketing is broad, ranging from the marketing of gambling websites to communication and advertising on the social media and networks. The objective of this article was to fill this gap by conducting a systematic literature review in order to answer the following questions: (1) What are the strategies of digital gambling marketing? (2) What is the effect of this exposure on gambling representations, intentions and practices?

**Method:** A systematic review was conducted following the PRISMA guidelines on Pubmed database (Medline) from February 2020 to March 2020 and Scopus. Existing papers published between January 2000 and February 2020 were identified by searching with this algorithm: (((“internet”[MeSH Major Topic] OR (communications[All Fields] AND media[All Fields])) OR (“social media”[MeSH Terms] OR (“social”[All Fields] AND “media”[All Fields]) OR “social media”[All Fields])) AND “gambling”[MeSH Major Topic]) AND (“marketing”[MeSH Terms] OR “marketing”[All Fields]), in title, keywords or abstract.

**Results:** Ninety-one candidate studies were selected, 21 studies were selected for the systematic review. Sport appeared as a specific target of online gambling marketing. A growing range of platforms for online sport betting and the development of strategies on the social media were identified. Regarding content, a systematic association between sport and gambling was highlighted. Vulnerable populations, such as young people, appeared to be at high risk of exposure to gambling marketing.

**Conclusion:** Little data is available on the strategies of digital gambling marketing or on exposure to it. Sport could be the first target for future research to understand how the industry is targeting specific populations, and what influence these strategies could have on PG development.

## Introduction

Internet has become a part of our lives and is both a medium for providing a wealth of information and an important tool for connecting with others around the globe (1). In recent years, the gambling industry has developed many types of gambling on different media, especially on the Internet. This expansion of legalized gambling has been identified as a public health concern (2–4). Gambling is a widespread social activity worldwide and nearly all national surveys conclude that there are more gamblers than non-gamblers (5). For example, 74% of the French population reported having gambled in their lifetime (6). In a majority of cases, gambling remains social gambling, but problem gambling (PG) can emerge (5). PG is defined as a persistent, maladaptive pattern of gambling resulting in clinically significant impairment or distress (7). Around the world, lifetime prevalence of PG ranges from 0.7 to 6.5% (5), and damage is severe: professional and financial (8), psychological, with an increased suicide risk (9), familial (5) etc. The trajectories of gamblers from initiation to PG development are influenced by many variables, including individual and environmental variables and also variables linked to the gambling characteristics (10, 11). Participation in gambling is increasing with the growing availability of gambling, advertising, marketing, and gambling deregulation (12, 13).

The gambling industry is one of the pioneers in internet technology development. It has designed gambling experiences to stimulate the human senses, by creatively integrating audio-visual technology, such as touch screens, surround sound, augmented reality, haptic actuators etc. (14). This strategy, based on experiential marketing, is very effective in influencing consumers' behavior, satisfaction, and loyalty (15). Through the creative use of touch, hearing and sight, the digital world has innovated in many ways of controlling and capturing human emotions (16). These evolutions in gambling types and the media used with the development of digital tools has enabled the gambling industry to expand its customer base (17). The legal status of online sports betting has been progressively changed and legalized in Europe since the mid-2000s, leading to a normalization of the practice. Consequently, the number of betting platforms legally available to consumers has increased. This has led to competition between companies to position themselves and attract customers to a relatively new market (18). Strategies developed by gambling operators on the internet can be included in the larger concept of the strategies of gambling marketing, defined as a management process from concept to customer.

Several studies have highlighted the links between the availability and proximity of gambling opportunities and excessive gambling practices (19–23). The causal mechanisms of the influence of advertising on gambling behavior are unknown despite a growing body of scientific evidence (24). Binde in 2014 in a critical review concluded that despite the lack of evidence, it was likely that gambling advertising had impact on gambling behaviors (25). Moreover in correlational studies, problem gamblers typically reported greater exposure to gambling advertising (26). Problem gamblers are a specific target for the gambling industry, in 2007, in Canada, 17.1% of online gamblers were considered as problem gamblers, and the money they spent amounted to 41% of the money spent online in the country (27). Gambling advertisements have been reported to have a greater impact on problem gamblers (25, 28, 29). Russel et al. found that in a large population of gamblers, 20% of those who reported a negative influence of repeated gambling advertisements were at risk or problem gamblers (30).

The recent prolific development in online gambling has been accompanied by growing concern for its potential harm (31). Regular and problem gamblers could be particularly concerned by the impact of digital gambling marketing. Online gamblers are defined as more at risk for problem gambling. Some studies have reported that online poker gamblers were two or three times more at risk of being problem gamblers than those gambling offline (27). In another study, Internet gamblers were significantly more likely to increase their gambling in response to online gambling promotions than non-interactive gamblers (26).

However, if advertising and traditional marketing have been reported to influence gamblers perceptions and behaviors, things are not as clear for digital marketing. Digital gambling marketing is broad, ranging from the marketing of online gambling websites to communication and advertising on the social media and networks. Social networks are considered to amount to a set of applications with various operating modes and uses: general networking (Facebook, MySpace), micro-blogging (Twitter), photo sharing, or exchange of ephemeral content (Instagram, Snapchat, etc.). These companies broadcast messages directly by insertion of classic advertisements into Internet users' news feeds, into stories, in the animation of official pages via community managers (Facebook, Instagram), and in the creation of cultural, sporting or festive events associated with the brand.

Analyzing the impact of the digital gambling marketing is important because 51% of people worldwide are connected to Internet (2019), especially young people: more than 90% of the 12 to 24-year-olds connect to the Internet every day, and respectively 80 and 94% of 12–17 and 18 to 24-year-olds used the social networks in 2019 (32). It can be supposed that the digital development of gambling and gambling marketing strategies on the Internet could influence gambling behaviors Very few studies in the literature have focused on this topic. The objective of this article was to fill this gap by conducting a systematic literature review in order to answer the following questions: (1) What strategies can be identified in digital gambling marketing? (2) What is the effect of this exposure on gambling representations, intentions and practices?

## Materials and Methods

### Protocol, Registration, and Eligibility Criteria

The PRISMA statement for reporting systematic reviews was adopted. Inclusion criteria were coded by both authors (MGL, KGM), reaching an agreement regarding the coding process and were as follows: (a) inclusion of studies concerning gambling marketing strategies on the Internet, (b) inclusion of articles containing quantitative and/or qualitative data, (c) inclusion of articles published in a peer-reviewed journal and following IMRAD, (d) inclusion of articles available as a full text in English or French.

### Information Sources and Search Strategy

From February 2020 to March 2020 existing papers published between January 2000 and February 2020 were identified by searching the academic databases Pubmed (medline), and Scopus. The two authors drew up a list of agreed English keywords for the systematic search: (((“internet”[MeSH Major Topic] OR (communications[All Fields] AND media[All Fields])) OR (“social media”[MeSH Terms] OR (“social”[All Fields] AND “media”[All Fields]) OR “social media”[All Fields])) AND “gambling”[MeSH Major Topic]) AND (“marketing”[MeSH Terms] OR “marketing”[All Fields]), in title, keywords or abstract.

The inclusion and exclusion criteria are presented in Table 1.

**Table 1:** Inclusion and exclusion criteria.

**Inclusion criteria**	**Exclusion criteria**
**Population**	
Gambling	Not concerning gambling
Internet or digital communications (phone, e-mails or text messages)	Not concerning Internet Concerning only “traditional media”: television, radio, newspapers or magazines.
All ages	
All types of digital marketing or advertising strategies (online gambling activities offered through interactive media, advertising on social media, pop-up ads, supported by the gambling industry or relayed by individuals)	
**Study design**	
Published in peer-reviewed journals, qualitative or quantitative studies or systematic reviews Following IMRAD format	Non peer-reviewed documents (e.g., websites, blogs, anecdotal evidence, case reports, guidelines)
**Countries, date, language**	
January 2000 - February 2020 Studies reported in English or French	In other languages

### Study Selection and Data Collection Process

The reviewers were the first two authors (MGL-KGM); they were researchers with previous experience in conducting literature reviews, and one of them had specific expertise in gambling disorders (MGL). The reviewers independently reviewed titles and abstracts, to ensure the reliability of the screening process. They then met to exchange their individual decisions and discussed their rationale for these decisions. Consensus was reached when the two reviewers agreed on article inclusion or exclusion. Full text articles for each included article were then collected, and screened by the two reviewers against the inclusion/exclusion criteria. The reviewers discussed any articles where a reviewer was unsure. Information extracted from the articles included: author names, year, and study location; journal, objective of the study, key results, key points of the discussion. Quality ratings were undertaken for all included peer-reviewed articles. We determined that all peer-reviewed research following IMRAD format was generally well-conducted and met the rating criteria. No studies were excluded for poor quality.

Ninety-one candidate studies were selected. After elimination of the duplicates (*n* = 7), and after reading the title and summary, 50 papers were retained after elimination of 34 studies(not concerning gambling marketing: 29, not concerning digital marketing: 4, not following IMRAD: 1).

After perusal of the full texts, 21 studies were selected for the systematic review, after elimination of 29 studies (not concerning gambling marketing: 13, not concerning digital marketing: 11, not following IMRAD: 5).

**Figure 1:**
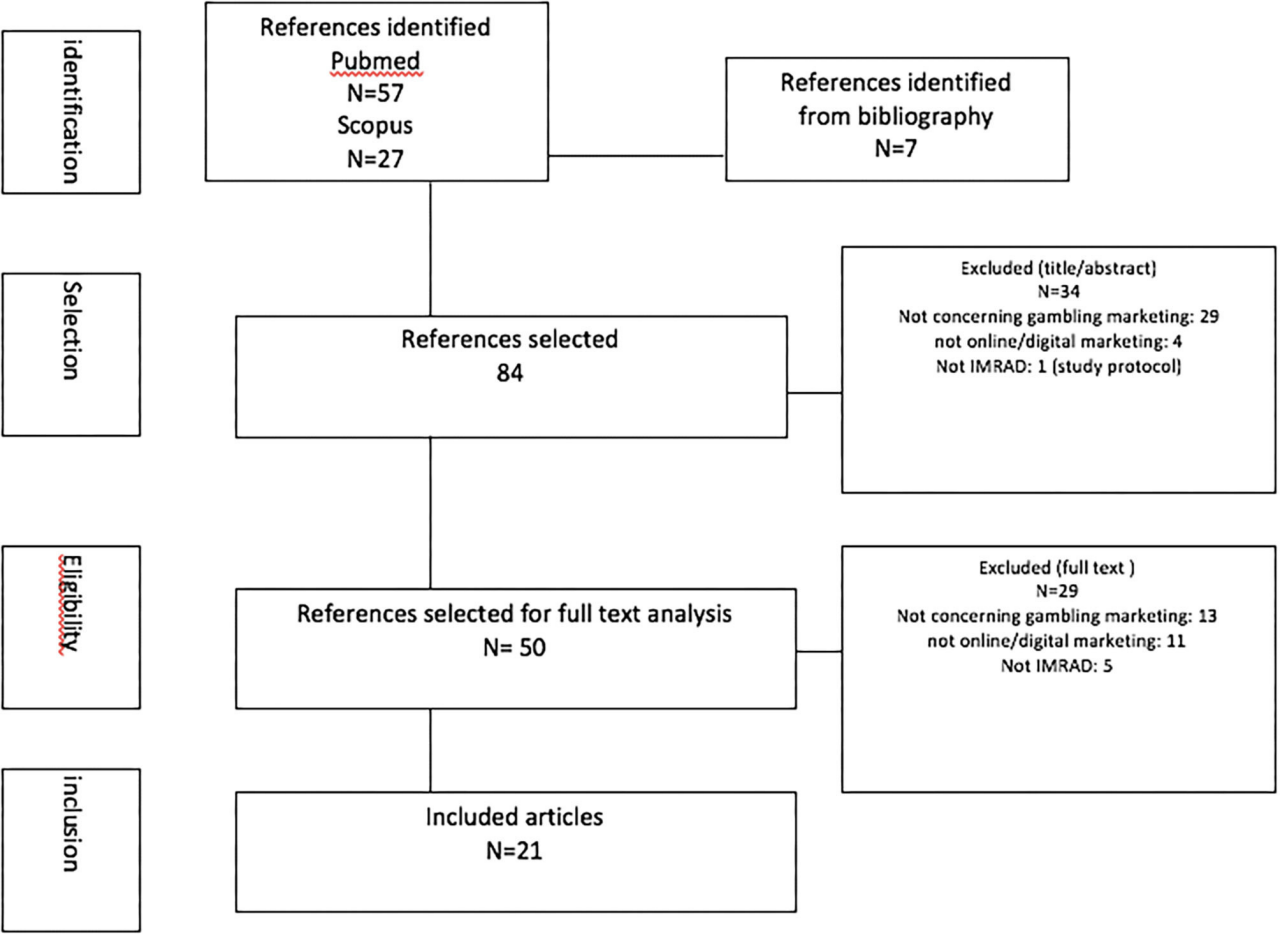
PRISMA flowchart.

The selection and inclusion processes are presented in a flow chart (Figure 1).

## Results

All 21 studies that met the inclusion criteria were analyzed. Of the 21 studies included, two were conducted in Europe (Spain, UK and UK) and one in Canada, one in USA/Australia and 17 in Australia or New Zealand. Quantitative methods were used in seven studies, mixed methods in five studies, qualitative methods in four studies, and content analyses in four studies. A majority focused on sport betting marketing strategies online (12 studies), only one study focused on poker, one on online bingo, and six studies concerned all types of digital gambling marketing strategies. One study concerned the marketing of social casino gaming. We included this study, because although casino games are free games, they are similar to gambling games. Users play with free virtual credits and cannot win monetary prizes, so that to some extent social casino games and gambling industry products converge (33).

Three main themes were identified in the selected articles. The first is that sport is a huge target for digital gambling marketing. A multiplicity of online platforms for gambling marketing diffusion have been identified and a wide range of digital gambling marketing strategies on the social media concerning sport betting have been observed. In addition, another recurrent subtheme was the systematic association of sport and gambling, fostering a normalization of betting and of gambling. The second theme was that digital gambling marketing strategies are gendered. A majority targeted young men, more particularly for betting and poker, and bingo websites were defined as targeting women. The third theme identified was that digital gambling marketing strategies focused on vulnerable populations, including young people and problem gamblers or at risk gamblers. The main results of the selected studies are presented in Tables 2A–D (2a: Articles concerning gambling marketing and sports; 2b: Articles concerning specific profiles (according to gambling characteristics: type of game, number of accounts); 2c: Articles concerning the use of social media or websites tools; 2d: Articles concerning harm reduction or responsible gambling and online gambling marketing).

**Table 2A:** Articles concerning gambling marketing and sport.

**Country**	**Author**	**Year**	**Journal**	**Objectives of the study**	**Type of study**	**Main results**	**Discussion**
Australia	Browne et al. (34)	2019	Journal of Behavioral Addictions	To determine whether exposure to betting advertisements and inducements influenced intended betting expenditure, or spending more than intended – and whether or not this differed by PGSI group	Quantitative study After completing a baseline survey, participants who were bettors (horse-racing or other sports) completed up to 15 short Ecological Momentary Assessment surveys: 5 per week over 3 non-consecutive weeks. The following were collected: participants' exposure to different types of betting advertisements, inducements, intended and actual betting behavior.	597 bettors completed at least one follow-up EMA survey *Exposure:* Horse-racing/sport bettors reported being often exposed to: -Gambling advertisements on betting app/websites: 14.0%/14.6% -Gambling advertisements not on app/websites not linked to gambling: 8.5%/8.3% -Direct messages: 11.1%/10.2% - Advertisements on social media posts: 11.1%/12.3% *Influence of exposure* Horse-racing bettors Exposure to company advertising, websites, and in-game commentary were independently associated with a greater likelihood of betting. Brand names and commentary were associated with increased spending, and with excessive spending. Inducements offered via direct messaging increased the likelihood of intending to bet, actual betting, and betting when not having intended to do so. Stake-back offers increased the likelihood of betting and the amount spent. Sports bettors Exposure to advertising on websites/apps and brand names, as well as to multibet inducements were associated with a higher likelihood of betting. Exposure to television advertisements was related to greater spending. Exposure to gambling websites/apps predicted an increased likelihood of betting when it was not originally intended.	The authors suggested that a reduction in betting advertising would be a positive consumer protection measure across the board. It would be likely to reduce betting expenditure and spending more than intended, including people at higher risk of experiencing gambling-related harm. Multibets and stake-back offers were the inducements that had the most influence on betting expenditure Direct messaging was a problematic form of gambling marketing: it was associated with a greater intention to bet, more betting, and betting more than intended for regular race bettors
Australia	Deans et al. (35)	2016	BMC Public Health	To provide a theoretical and empirical understanding of the use of symbolic appeal strategies in sports gambling advertising in Australia	Mixed method Analysis of the content of 85 sports betting advertisements issued by 11 Australian and multinational betting companies.	Ten main strategies appeared in the coding framework: Sports Fan Rituals and Behaviors/Mateship/Gender Stereotypes/Winning Social Status/Adventure, Thrill and Risk /Happiness/Sexualized Imagery/Power and Control/Patriotism Gendered messages were used in betting advertisements	The sports betting industry may be using multiple symbolic consumption strategies to influence social acceptance of sports betting, as used in the promotion of other unhealthy products The most overt strategy was the use of creative strategies to embed sports bets directly in sport rituals and practices, or to align gambling with peer-based social activities Sports betting advertising during sporting events or aligned with them was an exceptionally influential form of promotion The authors recommended new research on how processes of “symbolic consumption” are occurring and how marketing contributes to a new set of individual and peer group identities related to gambling on sport
Australia	Deans et al. (36)	2017	Harm Reduct J	To explore how marketing strategies can influence the gambling attitudes and consumer intentions of young men	Qualitative study, in-depth qualitative research with young male sports gamblers (20–37 years; *N* = 50)	Four main themes emerged: - Changing the marketing environment for sports betting products induces normalization: marketing reported in environments not designed for gambling (TV (100%), online (pop-up banners) (50%), and on social media websites (36%) and in gambling environments: on mobile sports betting apps 16%). - Participants described the role of sponsorship deals between the industry and sporting codes as creating a symbolic alignment between gambling and sports - The majority of participants believed that young men were the key target market for gambling companies and that marketing played an important role in shaping the gambling identities of young men - Many (*n* = 34) participants considered that the incentives offered by the betting industry were amongst the most effective marketing strategies in leading themselves and others to bet on sports.	Marketing for sports betting products is no longer confined to specific gambling environments. It has entered everyday community and media spaces. The “gamblification” of sports, has created a new cultural representation that betting is essential to the sporting experience. The authors recommended the development of sustained and adequately funded public education programmes, or mass media campaigns, developed independently from the gambling industry, to complement the legislative approaches already suggested for policy makers.
Australia	Hing et al. (37)	2017	Journal of Gambling Behavior	To examine whether responses to gambling promotions in televised sport vary with problem gambling severity amongst Internet sports bettors	Quantitative study Online survey of 639 sports bettors from Queensland, Australia	Male and younger online sports bettors had higher overall problem gambling severity than their female counterparts Significant predictors of higher PGSI scores were: being male, younger, more favorable sponsorship response, higher approval of gambling promotional techniques, and a higher subjective influence of gambling promotions on sports betting behavior	Internet sports bettors with higher problem gambling severity responded more positively to gambling promotions during televised sport. This study provided more detailed insights into how attitudes to particular aspects of sports betting advertising vary with problem gambling severity. Online sports bettors with more PG symptoms had a more positive response to gambling sponsors: increased awareness of, attention to, and recall of the sponsor's name and their promotions (interest), a more favorable disposition toward the sponsor (favorability), and a greater likelihood of using the sponsor's products (use). Attitudes that sports-embedded messages engender are more salient than frequency of exposure in predicting gambling problems amongst online sports bettors.
Australia	Hing et al. (38)	2018	Journal of Behavioral Addictions	To examine whether uptake of betting inducements predicts impulse betting on sport	Quantitative study Online survey on a panel of 1,813 gamblers (sports bettors)	More frequent uptake of all types of betting inducements predicted a more instantaneous, unplanned and unreflective approach to betting through the placement of in-play bets.	The authors concluded that more frequent users of sports betting inducements tended to bet more impulsively, but only in relation to impulse bets placed during the match. In-play betting *per se* was especially attractive to problem gamblers: it offers frequent, repetitive, gambling opportunities within a short timeframe. The promotion of betting inducements could increase in-play betting among problem gamblers by incentivizing and stimulating impulse urges to bet. The authors concluded that structural characteristics of betting products could lead to gambling problems. Respondents who reported lower incidence of seeing or hearing advertisements and promotions for sports betting when exposed to the media had a greater tendency to bet impulsively during play. The authors supposed that higher-risk gamblers were less consciously aware of this type of promotion when it occurred. The authors underlined that marketing targeted young male sports bettors, more impulsive and vulnerable to advertising.
Europe (Spain and UK)	Lopez-Gonzales et al. (39)	2018	J Gambl Stud	To examine the structural metaphors underpinning online sports betting (OSB) advertising and the consequences for bettors of this characterization	Content analysis of the structural metaphors underpinning OSB advertising in 135 advertisements extracted from YouTube channels of 29 betting brands	4 common reiterative metaphorical constructions were selected (out of 20 initially identified), betting was represented as: (1) an act of love: compared to love or friendly relationships, betting as an equivalent of showing your love to your team, the emotion of betting and winning a bet as compared to sex (2) a market: gambling is predictable, rational, and regulated and concerning professionals and experts; changes in betting amounts compared to stock price fluctuations (3) a natural environment: competition is akin to survival, bettor seen as a predator, intuition or superstition in betting seen as a natural instinct of animals, fast decision-making in gambling seen as ferocity (4) a sport: bettor compared to a manager, studying betting as training, in-play betting seen as playing, bet selection seen as a strategy, a winning bettor as a champion Absence of traditional gambling narratives: no dream metaphor. The multiplicity of forms that OSB advertising adopts accentuates the need for a neutral approach platform that analyses how betting activity is constructed in different settings	The most cross-sectional and enduring metaphor was “betting is a sport.” The notion of sport attached health attributes to brand names: success through work and skill, with possibilities of control over sports events, body consciousness, fat and sugar-free diet and exercise, team building, cooperation, joyfulness and amusement. This metaphor led to 2 interpretations: betting is understood as a sport and sport is understood in terms of betting. Advertising for OSB can use the emotional connections of bettors with their teams, athletes or organizations. The love metaphor appeals to gamblers' loyalty. The market metaphor reinforces the image of bettors as business people, controlling their risks, which is not the case, as bettors behave like fans, and betting marketing is increasingly advertising more complex bets with higher expected losses. The natural metaphor completes the market metaphor, this conflates the understanding of betting as an inevitable process that escapes individual volition, and is underpinned by the competitive backdrop in which bettors need to compete in order to win/ survive.
Australia	Macniven et al. (40)	2015	Health Promot J Austr.	To determine the extent of unhealthy food and beverage, alcohol and gambling sponsorship in Australia	Data collected from websites of the 53 national sport organizations (Australia) and 360 territorial sporting organizations; Structured survey tool assessing sponsoring content, classified as healthy or unhealthy, analyzed over 1 year (2012-2013)	1975 website sponsors identified. 26.9% of websites had only healthy sponsorships. 14.6% of sponsorship concerned gambling companies: - Australian football had the highest number of gambling sponsors. - Lottery West was the most common gambling sponsor.	Unhealthy sports website sponsorship is not consistent with the health-promoting goals of sport. The widespread unhealthy sponsorships pose ethical issues, such as the exposure of children. The statutory requirement for gambling companies to cede 5% of profits to the Western Department of Sport and Recreation, probably influences the presence of Lottery West on Western Australian websites, which is concomitant with branding presence. The authors concluded that sport was a very attractive venue for companies to reach people and promote products and brand names, but associations of unhealthy products with sport normalizes unhealthy products and undermines the health benefits of sports.
New Zealand	Maher et al. (41)	2006	BMC Public Health	To examine the extent and nature of both “healthy” and “unhealthy” sport sponsorship for popular sports in New Zealand	Quantitative study concerning sponsorship and type of sponsorship (healthy products vs. unhealthy products) of 107 sport organizations (belonging to the top eight sports for those aged 5–17 years)	73.8% (*n* = 79) of websites contained information about sponsorship Sponsorship of popular sports for 5 to 17-year-olds was dominated by sponsorship associated with unhealthy products, and by gambling in first place. Gambling was the most common specific sponsorship category: 18.8% of total sport sponsorship.	Sponsorship on popular sport websites in New Zealand was common. “Unhealthy” sponsorship was more than twice as prominent as “Healthy” sponsorship The authors concluded that governments may need to consider regulations that limit “unhealthy” sponsorship and/or adopt alternative funding mechanisms for sponsoring popular sports.
Australia	Pitt et al. (42)	2016	Aust NZ J Public Health	To investigate how children and adults recall the content and promotional channels of sports betting marketing	Mixed method study of 152 parent/child dyads (children 8 to 16-years old) conducted on Australian football League, national Rugby League, and soccer sporting sites in Australia	304 participants were included. 91.4% of the children and 98.0% of the adults recalled having at some time seen a promotion for sports betting. The top four environments for children were: TV (97.1%), stadiums (75.5%), radio (49.6%), and websites (46.0%) The top four environments for adults were: TV (96.6%), stadiums (61.7%), websites (45.6%), and newspapers (44.3%). 75% of the children and 90% of the adults perceived that sports betting was becoming a normal part of sport.	Children were widely exposed to sports betting marketing, for 46% on websites. Children were exposed to a range of industry tactics and reported that they regularly saw gambling marketing embedded in sporting programs, and they recalled gambling brand names. Children were more attuned to the content of gambling promotions than adults. Children specifically recalled promotions that fostered a perception of low risk or an increased chances of financial gain.
Australia	Pitt et al. (43)	2016	BMC Public Health	To explore adolescents' and parents' attitudes toward the marketing of gambling products in sport	Qualitative study conducted with 59 family groups (at least 1 parent and 1 adolescent 14 to 18-years-old) in Australia	Three main themes emerged *Initiation - the use of sport as a platform for the promotion of gambling* -Peak of gambling marketing during sports matches - Alignment of gambling with sports fan loyalty - Promotion of betting by sporting stars and commentators. *Influence - key promotional messages in sports-based gambling promotions* - An easy way to win money - Linking gambling to the emotion of the game - Linking technology to accessible gambling: parents perceived adolescents as being at risk because of the link between marketing and accessibility through mobile technologies and websites. Adolescent boys recalled they had seen marketing that talked about the ease of online gambling, provided incentives to open online accounts, and informed viewers how to access websites. They felt “encouraged to bet,” more particularly on the phone. - Intertwining gambling with the game *Impact - engaging in sport through a gambling lens* - An “everyday” part of sport - Discussing sport via gambling discourse	Parents and adolescents were aware of the increasing alignment of gambling and sport. Parents were increasingly concerned about the excessive promotion of gambling, in particular betting advertising in sport. They felt unable to counter the persuasiveness and volume of promotions of gambling. The authors concluded that policy makers should consider how they can expand regulatory frameworks to encompass a wider range of promotions that can occur outside traditional commercial-break advertising. Adolescents were aware of promotions outside traditional commercial-break advertising, they perceived that the use of current or ex athletes was an influential tactic in aligning gambling with sport.
Australia	Russel et al. (44)	2018	Journal of Behavioral Addictions	To determine whether betting expenditure is related to receiving direct gambling messages (text and e-mails), and the specific inducements they promote	Quantitative study Online survey in a population of bettors using a daily ecological momentary assessment The following were collected: numbers of emails, texts the participants received from betting operators; expenditure over the previous 24 h and intended over the next 24 h. Messages and emails were forwarded to the research team and analyzed.	98 sports bettors were included. They received an average of 3.7 emails and 2.3 texts over the course of 7 days. 104 horse-racing bettors were included. They received an average of 6.5 emails and 4.3 texts over the course of 7 days. Those who received more direct messages were more likely to intend to bet in the next 24 h, and for sport bettors to bet more money. The number of emails received was positively associated with both a higher intention to bet and intention to bet larger amounts, and likelihood of actually betting and the amount anticipated.	The authors concluded to a clear relationship between direct messaging from betting operators and both intention to bet, and actual betting behavior, including the amount bet. The channel used had an impact: emails were associated with intentions, and texts with actual expenditure. Direct messages, containing inducements or not, served as cue to bet. The authors suggested placing conservative limits on how frequently operators can message individual consumers, or requiring operators to only contact consumers with an account, and who have opted in, or to establish a “Do not” direct message.
Australia	Thomas et al. (45)	2018	Harm Reduct. J.	To enhance understanding of young people's exposure to and awareness of gambling advertising restrictions in Australia.	Mixed method Influence of online gambling advertising on young people (11 to 16-years-old) who were basket-ball fans	111 participants were included (mean age = 12.9 y.) *Engagement in sport* 97.3% played basketball for a domestic or representative team. 92.8% had watched professional basketball in the last 6 months: free to air and subscription TV (70.2%), via YouTube (23.4%), or other websites (15.3%) The 13 to 16-year-olds used social media to follow basketball players or teams via Instagram (52.3%), YouTube (21.6%), and Snapchat (21.6%). *Recall of placements and gambling advertising* Over 90% of young people reported seeing gambling advertising on television 55% of young people recalled seeing gambling advertising on social media platforms They saw gambling advertising at all times of the day, but particularly in the early evening before 8:30 p.m. 79.3% stated that there were too many gambling advertisements in sport and said there should be fewer or none.	Young people were exposed to gambling advertising across a range of different media platforms: TV and social media. The authors underlined that regulations focused on traditional media, like TV, but the social media are an influential marketing space for companies. The authors concluded that in Australia sport continues to be a large contributor to young people's exposure to gambling advertising. Most young people thought that sporting regulations should do more to protect them from exposure to gambling advertisements.

**Table 2B:** Articles concerning specific profiles (according gambling characteristics: type of game, numbers of accounts).

**Country**	**Author**	**Year**	**Journal**	**Objectives of the study**	**Type of study**	**Main results**	**Discussion**
USA/Australia	Abarbanel et al. (46)	2017	Policy Internet	To provide an empirical understanding of social casino gaming advertisements seen by young adults	Analysis of content of a sample of 115 social casino gaming advertisements captured by young adults during their regular Internet use	Imagery featured likely to appeal to young adults, with references to positive images (sport, cartoons, popular culture etc.) Messages included glamorization of gambling, winning, normalization of gambling, playing for free, and general encouragement to play 90% did not contain reference to problem gambling or responsible gambling	The authors recommended that: - Gaming companies recognize the potential harm of advertisements - Companies embrace corporate social responsibility standards: adding warning messages to advertisements and ensuring that marketing messages do not encourage excessive gambling.
Australia	Gainsbury et al. (47)	2015	Eur J Public Health	To compare online gamblers with a single Internet gambling account to those with multiple accounts	Quantitative study Online survey on a sample of online gamblers recruited through advertisements on various websites The following were collected: Internet gambling participation, and gambling-related problems	3,182 participants were included 45.2% had only one account *Gamblers with multiple accounts:* Participated in a significantly greater number of different forms of gambling Were more likely to do most or all their gambling online Were more likely to engage in sports betting, to classify themselves as professional, and as being moderate risk or problem gamblers Two-thirds were influenced by prices and gambling promotions in selecting gambling operators *Choice of website was based on:* For gamblers with multiple accounts: number of betting options, games available, fast payout rates, better interface For gamblers with a single account: advertising/marketing, jurisdiction where the site is regulated, whether the site is licensed, customer protection and responsible gambling tools.	Gamblers with multiple accounts were more involved (frequency, engagement in multiple activities). Advertising influenced those with a single account, but those with multiple accounts were more influenced by promotions: advertising was more influential in gamblers' initial decision to choose an operator. The authors supposed that gamblers with multiple accounts were willing to “shop around” to get their preferred experience.
UK	Stead et al. (48)	2016	PLoS ONE	To identify and analyse the characteristics of online bingo and explain the potential appeal of online bingo in the UK to bingo players	Qualitative study using 2 distinct data sources: content analysis of websites / in-depth interviews of 12 bingo players	*Websites* The bingo websites offered a wide variety of games and promotions, including big prize money, new member promotions and free games. All sites had information about self-exclusion. *Interviews: 3 themes identified* Drawing in the first-time user: sites presented as an exciting, likable and easily accessible experience. Bingo presented as normal, popular and ubiquitous. Gendered design of sites: color, hearts, cocktails, fashion glitter balls, offers for beauty products and references to “mums” Creating belonging: references to social interaction, inclusive language, community and chats on websites, development of feelings of belonging and cementing of relationships between the user and the game Stepping up involvement: users encouraged to include bingo in their daily routine, facilities offered to pre-purchase tickets for future games, use of metaphors such as metaphors of achievement, reward linked to engagement	Websites deployed a number of structural, textual and design features to draw in first-time users: easy to access, minimum age verification, possible to play and win “for free” before entering credit card details. The design, color, imagery of websites were designed to meet marketing objectives: the bingo websites had the effect of positioning online bingo as a benign, homely, women-friendly, social activity. Belonging was a major theme on the bingo websites, mascots and offers were used to convey a brand “personality” and to build a relationship between brand and users. There was congruence between the strategies used by websites and the motivations of bingo players: the bingo websites replicate and updated the sociability of traditional bingo halls. Online bingo differs from traditional bingo in its ability to be played anywhere, at any time, its capacity to offer a deeply immersive experience, and it is considered as presenting a higher risk of harm. The authors concluded that gambling marketing strategies influenced both new and existing players. Strategies used by websites performed 3 functions: drawing in new users, consolidating users' relationship with the websites by creating feelings of belonging, and encouraging existing users to step up their involvement.

**Table 2C:** Articles concerning the use of social media or websites tools.

**Country**	**Author**	**Year**	**Journal**	**Objectives of the study**	**Type of study**	**Main results**	**Discussion**
Australia	Gainsbury et al. (49)	2015	International Gambling Studies	To explore how gambling operators are using the social media to engage with users and promote products and services	Qualitative method Thematic analyses of 12 semi-structured interviews with 19 individuals representing different sectors of the gambling industry	*Use of the social media* The social media are integrated into a global strategic business and communication plan, with the aim of increasing brand-name awareness and customer commitment Facebook, Twitter, YouTube, Instagram and Pinterest were used by operators. Community narratives are an important part of the social media content. *Target audience* Operators targeted young men. The social media were considered useful to engage with new consumers by enhancing brand-name salience, building customer relationships, and encouraging visits to their website *Impact of the social media* Risks of negative feedback for brand names were cited: companies have no control over how consumers engage with the company. Risks of reputational damage were noted *Inclusion of responsible gambling* Most operators stated that they included responsible gambling messaging in the content posted.	The social media involvement appeared to be crucial for gambling operators and is increasingly embraced The social media were used to engage with existing customers, and potentially reach users already interested in gambling products. Successful use of the social media was measured from brand involvement. The goal of increasing sales was not reported by operators. The social media were defined as a way to recruit customers by different means: running competitions, asking questions, posting relevant articles, links and stories, or responding to customers' comments, queries or complaints All operators appeared to be mindful and cautious about ensuring that the social media were not used to promote excessive gambling and did not target vulnerable populations (consistent with Australian advertising rules of conduct). But little control of the sharing of contents with minors. Several operators included responsible gambling messages on their social media profiles, but most of the time, they were not accessible. Operators found that the social media were not an appropriate channel for discussion on responsible gambling and that users would not like these messages.
Australia	Gainsbury et al. (50)	2016	J Gambl Stud	To examine the use of the social media for marketing purposes by gambling companies	Audit of 101 sites over 4 weeks: Mixed method, quantitative variables collected and thematic analysis of social media utilization by gambling operators in Australia	*Quantitative data* 87% of operators had a Facebook page, 52% a Twitter page 11.9% of operators had information about responsible gambling or PG services on their social media profiles *Qualitative data* Latent message promotional content: raising awareness/glamorizing gambling/emphasizing ease of use/Encouraging new use/emphasizing winning/encouraging venue patronage/encouraging betting/aligning gambling with sport/brand engagement/promoting community benefits of gambling/limited warning messages	The majority of gambling operators had social media presence, betting agencies more particularly The most popular social media platform was Facebook Gambling was depicted in an overwhelmingly positive light: glamorous, exciting, fun Gambling promoted as having a natural alignment with sport to convey gendered messages: gambling a way to show masculinity, team loyalty, skills etc. The practices of gambling operators encourage potential sharing of social media posts, facilitate exposure of vulnerable populations to gambling marketing (underage individuals) A lack of responsible gambling content on social network pages and content posted by Australian gambling operators The authors recommended research to monitor the impact of gambling marketing via the social media on young people
Australia	Gainsbury et al. (29)	2016	Psychol Addict Behav	To investigate recall of exposure to, and reported impact on gamblers of gambling promotions on the social media, with a focus on current problem gamblers	Online quantitative study on a sample of 964 participants (self-reported use of social media and gambling within the previous 12 months)	*Exposure* Moderate-risk gamblers significantly more likely to report having seen gambling promotions on the social media than non-problem gamblers (66.2 vs. 39.8%), and to report having seen all types of gambling promotions, and having interacted with gambling operators on social media. *Impact of exposure* 29.3% of moderate-risk gamblers reported that social media promotions had increased their problems. A minority of low-risk and non-problem gamblers reported that their gambling had changed under the influence of promotions. *Responsible gambling messages* The majority did not recall seeing responsible gambling messages on social media. Moderate/risk-prone gamblers were significantly more likely to recall responsible gambling messages on social media websites than non-problem gamblers.	The authors distinguished a subset of vulnerable gamblers for whom social media marketing could influence their gambling problems: better recall of promotions, and reported influence on gambling practices. The authors suggested that operators were not as vigilant at detecting users with gambling problems as claimed, or that it is difficult to detect gambling problems on the basis of social media interactions. The authors concluded that moderate-risk gamblers were an appropriate target audience for responsible gambling messages, and were more receptive to the use of social media platforms.
Canada	McMullan et Kervin (51)	2012	Int J Ment health and Addiction	To examine the web design and engineering of advertising and marketing, and pedagogical features present in a random sample of 71 international poker websites obtained from the Casino City directory in the summer of 2009	Qualitative study Content analysis of 71 poker websites	22 variables were coded, related to access, appeal, player protection, customer services, on-site security, use of images, text, language, interactive and immersive materials, promotional products and programs, sponsorships, celebrities, tutelage resources, responsible gambling programs	The poker websites were defined as an instrument of friendship. 92% of the websites defined poker as a natural consumer activity. Poker websites were instruments of promotion: attractiveness, bright-color, design, 97% used promotional sales practices, 81% featured reward programs and 76% affiliate programs. Marketing targeted young people: 28% of the individuals portrayed in images appeared to be 25 years old or under Gendered marketing strategies were identified: 11% promoted overtly sexualized images to send the message that poker was seductive

**Table 2D:** Articles concerning harm reduction or responsible gambling and online gambling marketing.

**Country**	**Author**	**Year**	**Journal**	**Objectives of the study**	**Type of study**	**Main Results**	**Discussion**
Australia	Howe et al. (52)	2019	Plos One	To determine the relative importance of selected predictors (including the degree to which individuals see advertisements and receive promotional material) in determining both gambling frequency and PG	Quantitative study using an online survey panel	3,361 participants were included *Factors associated with gambling frequency* The degree to which peers or family were perceived as gamblers, self-reported approval of gambling, participation in offline discussions on gambling, PGSI scores *Factors associated with PGSI scores* Exposure to advertisements and receiving promotional material were correlated, but 91% of the explainable variance could be explained by 5 predictors: positive urgency, playing on poker machines at pubs, hotels, or sports clubs, gambling on the Internet, online discussions on gaming tables at casinos, overestimating chances of winning.	The degree to which others being perceived as gamblers was one of the strongest predictors of gambling frequency. Individuals overestimated how much others gambled and overestimated how far they approved of gambling. The authors suggested that interventions designed to reduce PG should concentrate on identified factors: reduction of access to poker machines, interventions to reduce people's overestimation of their chances of winning. A campaign of this type could aim to educate people to avoid common gambling fallacies.
Australia	Thomas et al. (53)	2017	Harm Reduct J	To explore how Victorian adolescents and adults attribute harm to different types of gambling activities To examine the extent to which Victorian adolescents and adults support the introduction of strategies aimed at reducing the harm associated with gambling	Mixed quantitative and qualitative method study Online panel survey to explore the attitudes of 500 Australian residents (16 to 88-years-old)	500 participants were included *Gambling practice* 40.2% of participants were at risk of experiencing some level of harm from gambling (PGSI ≥ 1) 16.6% recorded scores that indicated problem gambling (PGSI ≥ 8) *Perception of harm* The mean level of perception of harm was higher for casinos and EGM than for horse-racing or sports betting. Participants defined characteristics entailing risks of harm according the type of gambling: Casinos: seductive nature of the venue, no concept of time, environment encouraging gambling. EGMs: perception of EGMs as deceptive or exploitative, perception that EGMs were not risky, accessibility and availability Horse-racing and sport betting: multiple markets offered by online betting providers, constant availability of opportunities to gamble, easy to lose financial control when betting on apps, and role of marketing in the normalization of sports betting. *Agreement and disagreement with gambling harm reduction strategies* More than 90% of participants agreed or strongly agreed with a ban on gambling advertising during children's viewing hours (*n* = 457, 91.4%) 86.2% of participants agreed or strongly agreed that sporting organizations should take more responsibility for how gambling is promoted. There was strong agreement with proposals for increased public education about the harm associated with gambling.	EGMs and casinos were identified as the most at risk, participants aware of EGM risks Perceptions of harm do not necessarily translate into behavioral choices. Overwhelming community support for: - Campaigns that focus on educating the community about the harm associated with gambling - Stricter boundaries placed around gambling products and the marketing of these products Government approaches in Australia are out of line with community attitudes and public expectations for mechanisms to protect communities from potentially harmful products. The authors sound a caution, in case of significant efforts of regulation of products, and negative community attitudes, industries could develop counter-measures to appear as “good corporate citizens” to avoid or minimize the impact of restrictions or regulations.

## Discussion

This review included only 21 articles on the topic of the digital marketing of gambling. They were for a large majority conducted in Australia or New Zealand. This lack of data, more particularly for North America or Europe, is surprising, given the development of online gambling and online internet gambling marketing in the last 10 years. As an example, the total market value of the global mobile phone gambling industry increased 10-fold between 2006 and 2011 ($23 billion compared to $2 billion) (54) These developments, and the structural characteristics of Internet, combining easy and cost-effective access, has prompted the gambling industry to widely invest in emerging technological tools. The high level of exposure to positive gambling cues in society has led to the perception of gambling as an acceptable, credible and harmless leisure activity (55).

### Sport: A Huge Target for Digital Gambling Marketing

In the literature on Internet marketing of gambling, the main emerging area concerned sports betting. The majority of selected articles (12) concerned gambling marketing in relation to sports.

#### A Multiplicity of Platforms and the Development of Strategies on the Social Media

The multiplicity of online platforms has enabled both the development and the repetition of positive messages promoting gambling practices and brand-names.

Gambling advertising has entered everyday life, and people can be exposed without having sought tony information on gambling. Gambling advertising and promotions can be found outside the traditional commercial-break advertising (43). Deans et al. showed that gambling marketing products had entered everyday community and media spaces. In their sample of young men, 50% reported having seen online betting marketing (pop-up banners) and 36% had seen it on the social media (36). In a qualitative study, Pitt et al. showed that parents and adolescents were conscious of the increasing development of marketing, more particularly for sports betting. Parents thought their adolescents were at risk because of the link between gambling marketing and accessibility via mobile technologies and websites (43). Browne et al. using an Ecological Momentary Assessment found that more than 8% of bettors remembered exposure to gambling advertisements on unrelated apps or websites. More than 11% reported social media posts concerning gambling and more than 10% reported direct messages. This last strategy is a specific concern: direct messaging via e-mails, texts, and phone calls from gambling operators is a problem. The majority of these direct messages promote specific gambling inducements, and bettors report that this type of marketing is intense and particularly influential on their betting, encouraging them to bet and to spend more on betting (44). Browne et al. also found that this type of advertising was associated with greater intention to bet, more betting, and betting more than intended for regular horse-race bettors (34).

The digital media have helped to broaden the scope of advertising messages, especially in sports betting. Gainsbury et al. showed that in a large sample of online gamblers, online gambling advertising influenced gamblers in their initial decision to choose an operator. They also reported that those more involved, with multiple online accounts, were more active bettors and were influenced by promotions (47). Browne et al. showed that exposure to gambling marketing increased the likelihood of betting, and increased spending on bets. They concluded in their study that gambling marketing negatively affected substantial numbers of bettors already at risk for, or currently experiencing, gambling problems (34). The promotion of gambling inducements increased impulsive in-play betting among problem gamblers and involved gamblers at higher risk of problem gambling. They were however less aware of online gambling promotions, compared to less involved gamblers (38).

Regarding the social media, the prevalence of users of the social media in the world is high, particularly in higher-income countries such as North America, where 56% of the population are active social network users, or in Western Europe, where 43% are concerned (50). The social media enable gambling operators to promote products and brand-names with fewer constraints than in traditional forms of media. Many social marketing campaigns aim to generate the equivalent of “word-of-mouth” (56). Social media marketing strategies have the potential to create a particular personal relationship between users and brand-names (57). Research on brand engagement on the social media has found that relationships between consumers and the brand-name, the product and companies all positively influenced trust and brand loyalty (58). An Australian study has shown that reputation is the most important factor in choosing an online gambling site (47). Even a limited use of social media by gambling operators could have a large impact in terms of promoting gambling products and causing harm. Through the social media, gambling marketing reinforces social norms and over-represents attitudes among fans, followers and their peers (50). The social media are used to portray a “brand personality,” and to foster enthusiasm in their communities (49, 59). Interviews of gambling operators have suggested that the social media are perceived as useful tools to increase website traffic, to raise interest and awareness and ultimately to increase gambling sales (60). Gambling operators are established on the social networks, Facebook and Twitter, collecting an average of 62,084 likes and 30,594 followers across the UK's top 10 betting sites (61). A survey of Australian gamblers found 40% had seen gambling marketing on Facebook (28).

#### A Systematic Association of Sport and Gambling Fostering a Normalization

The extent of gambling advertising and penetration through the digital media and Internet is a contributory factor in strengthening the mental association between sport and gambling (62, 63). The content of gambling advertising reinforces links between gamblers and sport: betting is rooted deep in the relationship between sport and fans (39). For example, the love metaphor is used in gambling advertising online, calling on both romantic love and friendship, and appealing to bettor loyalty. Gambling is depicted as a truly positive activity. On the social media, some posts portray gambling as glamorous, exciting and fun, others emphasize gambling winnings, and community benefits are also highlighted (35, 50). The message conveyed through these positive contents is that gambling provides easy money, fun, enjoyment and an entertaining, easy, effort-free lifestyle (64). The sports betting industry uses numerous symbolic strategies to promote the social acceptance of sports betting, similar those to used in the promotion of other unhealthy products, such as alcohol or tobacco (35).

Gambling marketing influences gambling perceptions and interpretations of gambling and minimizes the risks. One of the main and longer-lasting effects of gambling advertising is the normalization of gambling (65, 66). Normalization is a long-term process, including sub-processes of cultural and legal legitimization. Gambling marketing cues introduced into the community and daily life (36) normalize potentially risky products by portraying their use in different everyday situations. Gambling marketing attempts to elicit emotive responses, or to trigger memories (50). Some author have referred to “the sportification of gambling and the gamblification of sport” (67). This phenomenon is identified in different articles: Gainsbury et al. found that the aligning of gambling with sport was a frequent content, and Lopez-Gonzalez showed that engagement and loyalty is also used to enhance involvement in gambling (39, 50). The risk underlined by authors regarding this association between betting and sport is that sport is represented as systematically associated with gambling, while gambling is represented as a sport (35, 36, 39). Online sport advertising uses the metaphor of betting as a sport, and the gambling companies are thus associated with the healthy attributes of sport (39). Moreover, if gambling is a sport, skills and training could help gamblers to improve their results, and these messages could reinforce cognitive distortions among gamblers, which is one well-known risk factor for problem gambling (68, 69).

Sport is a very attractive venue for companies to reach people and promote products and brand-names (40). Sponsorship of peak sporting events by unhealthy food, beverage, alcohol, and gambling product companies is prevalent in Australia according to the results of Mc Niven et al. who reported that 14.6% of unhealthy sponsorships concerned gambling (40). Sport sponsorship is a marketing tool, more acceptable by the public because it is indirect and it builds public goodwill toward the company (70). It associates sponsored products with a healthy positive image, which is particularly important for products that can involve risks for health (70). A study conducted in 2006 by Maher showed that gambling was the first sponsorship product in the most popular sports for 5 to 17-year-olds in New Zealand (41). In 2015 Macniven showed that only 26.9% of national sport organization websites had solely healthy sponsorships, and that 14.6% of sponsorships of websites concerned gambling companies.

These strategies concerning sport and online gambling have been implicated in the general development of gambling. One study showed that gambling advertising was associated with the development of sports betting among people who did not previously gamble (71). In a recent study, Newall et al. in the UK analyzed “Live-odds” gambling adverts, during World Cup matches on TV. They showed that advertisements were skewed toward complex events, more difficult to predict, and that the content of advertisements made bets appear more urgent than necessary (72). With this development and potentially greater diversity in gambler populations, there is likely to be an extension to new population groups experiencing problem gambling, and greater concern for vulnerable populations.

### A Gendered Marketing Strategy

Young men are defined as targets for betting and poker websites. Australian gambling operators interviewed by Gainsbury et al. reported that, on the social media, they targeted the population of young adult men (49). In an exploratory study of gambling operator contents, the same authors showed that gambling was naturally aligned with sport, to convey messages that gambling is a way to demonstrate team loyalty and masculinity (50). Deans et al. showed that young gamblers believed that young men were especially vulnerable to gambling harm, and that marketing amplified the risks associated with sports betting and played an important role in shaping the gambling identities of young men (36). In another study analyzing the content of sports betting advertisements, Deans et al. (35) showed that there was clear gender stereotyping in sports betting advertising. Men were mostly represented as central actors, women were sexually objectified, with advertisements portraying male dominance or power over women. Two key stereotypes of men in Australian gambling advertisements were noted: the first is the average “Australian male,” for whom sports gambling could represent an escape from the ordinary to become more attractive to women, to gain power and authority or to be able to afford a glamorous lifestyle. The second stereotype concerns bookmakers, portrayed as powerful players (35).

Regarding the online sports betting marketing, metaphors are used, and among four metaphors identified by Lopez-Gonzalez et al. the metaphor of “gambling as a market” and “gambling as natural” could also be compared to a gendered approach. These metaphors represent betting as an inevitable, innate behavior, akin to instincts or sexual relationships. Betting is defined as an inevitable process, escaping individual volition, as a survival process or as a struggle to survive (39). These gendered digital marketing strategies are particularly concerning, as young adult males are the socio-demographic group the most at risk for gambling problems (11). Hing et al. showed that impulse betting both before and after match commencement was more frequent among young men, who were clearly the target for sports betting advertising, including promotions for incentivized bets during play (38). Concerning poker websites, marketing strategies were also shown to be focused on men: Mc Mullan and Kervin analyzed online poker websites and found that adult-oriented imagery, such as young women in bikinis or adults depicted in sophisticated clothing and settings, were frequently used (51).

In contrast, one study conducted in the UK on bingo websites, showed that marketing strategies on these websites were congruent with the expectations of women who play bingo. The authors reported that bingo websites seemed to be designed largely to appeal to women, through the use of the colors pink and purple, images of hearts, cocktails, fashion, and glitter balls, offers for beauty products, and references to “mums.” The bingo sites had the effect of positioning gambling as a benign, child-like, homely, women-friendly, social activity (48).

### Online Gambling Marketing and Vulnerable Targets

In a public health approach to prevention of gambling and in order to determine the potential impact of gambling marketing on vulnerable populations, a comparison can be made with alcohol. Babor et al. established that young people and heavy drinkers are vulnerable populations for exposure to alcohol marketing strategies (73). The same vulnerabilities can be presumed concerning gambling behaviors and gambling marketing methods. An early age of initiation is a high risk factor for the development of problem gambling later in life, and it is associated with greater severity of problem gambling (74–76). Despite the fact that regulations prohibit gambling by minors in many countries, for instance France or Spain, evidence exists that these populations gamble (11, 77). Gambling advertisements and specific promotions also have a greater impact in encouraging gambling amongst problem gamblers than among non-problematic gamblers (78).

#### Younger Targets

The familiarity of minors with the Internet increases their likelihood of playing. For instance, 72% of adolescents use the Internet more than once a day in Australia (79). Pitt et al. showed that 8 to 16-year-olds were widely exposed to sports gambling marketing, for 46% through websites (42). In addition, online gambling is private and feasible anywhere, and online gambling websites offer prizes and a wide range of temporary promotions. Online gamblers report a positive playing experience and greater physical comfort than offline gamblers (80). In another study adolescents felt “encouraged to bet,” more particularly on mobile phone (43).

Major social media and online gaming companies have started making inroads into the gambling business. This “digital convergence” has created opportunities for the gambling industry to expand its customer base, particularly among young people (81). The evolution of technical aspects of betting, such as opening accounts and betting via mobile phones, are also perfectly modeled for young people (82). The proliferation of simulated forms of gambling not involving money on the social media is a gateway to encourage adolescents to progress to online gambling. Social gambling can also lead to a diversification of gambling offers for young people, with an easier, more attractive access to casinos. Abarbanel et al., using a content analysis of a sample of 115 social casino gaming advertisements, clearly showed that the images and messages were designed to target young populations, by including references to popular culture, cartoons, and sport, and deploying a glamorization and encouragement for gambling, including free play (46).

Despite this observation that young people are particularly prominent consumers on digital media platforms, very few studies have focused on this topic (45, 83). There is still very limited information about the promotion of gambling on these media and on how it influences the exposure of young people to gambling advertising (45, 83). Deans et al. interviewed a sample of young male gamblers, and the majority believed that young men were the key target for gambling companies (36).

However, digital communications are liable to enhance exposure to favorable presentations of online gambling. An analysis of poker websites showed that 28% of the images portrayed concerned people aged 25 or under, in attractive environments (51). Gambling marketing clearly influences gambling intentions. Derevensky et al. noted that 40% of the young people in their study stated that they had wanted to try gambling after seeing gambling advertisements (77). Thomas et al. found that 75% of a sample of 8 to 16-year-olds could recall the brand name of at least one sports betting company (84).

Many European countries have identified a large increase in gambling participation among underage young people. For example in the United Kingdom, 38% of the 16 to 24-year-olds gambled in 2016 compared to 5% the previous year (85). In other reports, ~60–80% of young people engaged in formal or informal gambling before the legal age (11, 86, 87). This population is at higher risk of losing control compared to older adults, and the prevalence of problem gambling is higher. In Finland a survey identified 4.9% of 12 to 15-year-olds as risk-prone gamblers (88); in Sweden one study found that the incidence of PG among 16 to 24-year-olds was more than double the proportion for adults aged 25–44 years (89). Links between the development of marketing strategies, more particularly online, and these gambling behaviors among young people need to be explored further. Gainsbury et al. for their part failed to show that content on social media directly appealed to young people. However, given the few restrictions on social media use, the inherent difficulties in monitoring and the widespread use of social media among young people, continuing research is needed to monitor the impact of gambling marketing via the social media on young people (50).

#### Problem Gamblers

Hing et al. in an online survey on a sample of 639 online sport bettors in Australia, showed that attitudes to particular aspects of sports betting advertising vary with PG severity. Online sports bettors with more severe PG symptoms had a more positive response to gambling sponsors: increased awareness of, attention to, and recall of the sponsor's name and their promotions (interest), a more favorable disposition toward the sponsor (favorability), and a greater likelihood of using the sponsor's products (use) (37). The frequency of gambling on the Internet and participation in online discussions on gaming tables at casinos were predictors of gambling severity in a study by Howe et al. (52). Moderate-risk gamblers were significantly more likely to report seeing gambling promotions on the social media, and nearly 30% of moderate-risk gamblers reported that social media promotions had increased their problems (29). Gambling advertising compromises gambling prevention campaigns aimed at reducing gambling and encouraging help-seeking. The positive messages on gambling conveyed through the social media are not counterbalanced by warning messages, as observed by Gainsbury et al.: only 11.2% of the operators had information on responsible gambling or problem gambling on the social media (50). Moderate and risk-prone gamblers are more attentive to responsible gambling messages (50). Thus, given the impact of social media marketing on vulnerable gamblers, the inclusion of responsible gambling messages on these platforms seems effective (50). In addition, social media marketing influences both infrequent and frequent gamblers, who may be unable to resist urges to gamble elicited by external cues found in advertising (90). Gainsbury et al., in a study including 2,799 gamblers, found that problem gamblers were significantly more likely than non-problem gamblers to be influenced by promotions and incentives, such as credits or bonuses provided by online gambling sites (78). However, many difficulties exist in the development of responsible gambling messages. Aspects that are critical to the effectiveness of these messages concern the type of content used, the way it is framed, whether it engages consumers in self-referential processing, the level of specificity and applicability for use in real-world settings, and the social norms deployed. Messages should be personalized to target specific population subgroups. Adequate understanding of the characteristics of these subgroups is important and could enhance the presentation of health information (91).

### Implications: A Need for Regulations?

Over the last 2 decades there has been a significant shift toward more liberal gambling regulatory frameworks in many countries around the world. The availability and accessibility of gambling has risen in community settings. The Internet has evolved rapidly, leaving policy makers and regulators far behind the innovative commercial products and offers (92). More recently, the liberalization of gambling has led to a legalization of more pervasive forms of gambling, alongside the development of new technologies and higher-intensity products leading to a larger penetration of gambling products in the community (53).

Governments have been largely unwilling to enact a comprehensive public health approach to gambling as applied in other areas such as tobacco. Governmental regulation efforts remain focused on individual responsibility frameworks to minimize the harm associated with “problem gambling,” which place few constraints on commercial activities and enable continued increases in revenue for both industry and government. There is growing ethical tension for governments between the revenue obtained from gambling products, and the need to be responsible and design rules that are acceptable for the community and public health (93).

It is important that regulations should keep pace with the advances in technology to ensure that social media platforms fall under the same regulatory frameworks as traditional advertising channels (45). Indeed, existing regulations do not apply to gambling advertising on social media platforms. This includes promoted content on YouTube, Instagram or Snapchat, which are the three most widely used social media by young people (45). To protect consumers better, any restrictions should cover digital as well as traditional advertising, to prevent the migration of advertising to less restricted, online, social media, and mobile platforms, as has occurred with the introduction of earlier advertising restrictions (34, 49). As online gambling companies should be responsible for the harm related to their activities, Yani-De-Soriano suggested that corporate social responsibility policies should be fully implemented, monitored and clearly reported; all forms of advertising should be reduced substantially, and unfair or misleading promotional techniques should be banned (94). Gainsbury et al. found that gambling operators reported being cautious toward the risk of problem gambling, but that social media operators thought they were not suited to discussing responsible gambling (49) and most operators do not incorporate responsible gambling into the content posted (50). In many countries and particularly in Australia, as identified in this literature review, regulations have predominantly focused on traditional media such as television, and there are no regulations to restrict gambling advertising on social media platforms. In the UK, there have been some attempts to enforce restrictions on gambling advertisements online, with the banishment from websites of gambling advertisements directed toward young people (95).

It has been shown in Australia that there were discrepancies between government regulations and public expectations. Government approaches were not in line with community attitudes and public expectations for mechanisms for protecting communities from potentially harmful products (53), even for young people (45). Abarbanel et al. in a sample of social casino gaming advertisements targeting young gamblers, showed that 90% did not refer to responsible gambling or the risk of problem gambling (46). Thomas et al. reported that young people thought that sport regulations should protect them better from exposure to gambling advertisements. Young people reported a need to remove gambling advertising from sport (45). Targeted problem gambling prevention could be developed, and Gainsbury et al. hypothesized that moderate-risk gamblers were an appropriate target audience for responsible gambling messages and were more receptive to the use of social media platforms (29). Community support for advertising restrictions is much stronger than for other harmful products (such as alcohol or tobacco) (53). In another study, more than 90% of the participants agreed or strongly agreed with a proposed ban on gambling advertising in Australia (53).

However, caution is necessary regarding regulations. First, statutory requirements for gambling companies could in fact enhance gambling sponsorship, as in Australia, where 5% of the profits of West Lottery are due to the Western Department of Sport and Recreation. This probably influences the presence of Lottery West on Western Australian websites, ensuring brand-name presence (40). Thus, in the case of regulations limiting “unhealthy” sponsorships, governments would also need to adopt alternative funding mechanisms for sponsoring popular sports (41).

Petticrew et al. showed that the gambling industry, like the tobacco, alcohol, or food industries, frequently uses the concept of complexity, in response to policy announcements and to new scientific evidence. “Complexity” is apparently used to distract the audience from the industry's contribution to the problem and to promote inaction or ineffective solutions (96). When there is significant support for the regulation of products and negative attitudes in the community toward industries such as gambling or alcohol and tobacco, those industries could develop new strategies or countermeasures. For instance they might frame themselves as “good corporate citizens” to avoid or minimize the impact of restrictions or regulations (53). Some governments and government agencies periodically attempt to counter pro-gambling messages, for instance the Victorian Responsible Gambling Foundation which promoted a social media campaign named “Love the game, not the odds.” However, it is hard for these transient social media campaigns to counteract the overwhelming pro-gambling messages (97). Media campaigns that emphasize the damage associated with gambling reduce gambling intentions, but pro-gambling media campaigns are much more effective in enhancing intentions to gamble (98, 99).

It will be important for public health advocates and coalitions to consider and recognize these strategies and to develop adapted online gambling regulations (100).

### Future Research Development

Gaps in the literature were identified here and could fuel future research. Beyond the evaluation of influence and content analysis, there is no data on the exposure to digital gambling marketing stimuli, in terms of modalities, frequency, time, or potential influence. Secondly in the case of digital alcohol marketing, participatory forms generated by users but driven by the industry's marketing have been described (101). These strategies mobilize intermediaries (influencers) who disseminate messages in favor of the industries within the framework of remunerated partnerships. In addition, industries also encourage Internet users themselves to interact with the official pages of their brands (follow, like, comment, identify a friend, share, re-tweet, etc.) via the humorous content of quizzes and riddles, or contests. There is little data on the influence of these strategies in the context of gambling. There is also little research on the impact of gambling advertising online, on inducements or on loyalty programs (102).

Finally, regarding social interactions and the diffusion of gambling behaviors, the social media afford new opportunities for intervention, such as online counseling or pop-ups that remind users of the time and money spent on gambling. Embedded messages in sports contents are more salient than frequency of exposure in predicting gambling problems amongst online sports bettors (37). This implies a need for social marketing and public education to counter promotional messages. They should aim to moderate positive sentiments toward gambling, brands and their promotion, since this is what that leads to excessive gambling. Social marketing is still a largely unexplored avenue for the prevention of gambling, and more particularly among young gamblers (103).

### Strengths and Limitations

This study focused on gambling, a growing public health concern, for which a preventive, therapeutic approach is needed. Twenty articles were selected following PRISMA guidelines among 64 identified initially. The analysis of these articles enabled identification of themes and characteristics of digital gambling marketing. One limitation is the focus on only two databases (Pubmed and SCOPUS), which could limit the results. In addition, the results of this review are subject to two biases limiting the generalizability of the data. There is firstly a cultural bias, in that a majority of studies concerned Australia or New Zealand. There is also a selection bias since a majority of the studies selected focused on digital strategies in sports betting. We did not include studies concerning gambling marketing on traditional media (television, radio, press).

## Conclusion

The literature is currently sparse regarding digital gambling marketing, despite its huge development in recent years. The main available data concerns the development of digital marketing and sports betting, and their vulnerable targets, especially young people. We have shown in this review that sport is a major target for marketing, and operators have developed gendered marketing strategies to reach and influence gamblers' behaviors. The multiplicity of forms that online gambling marketing and advertising adopt accentuates the need for research on content and exposure on digital platforms. This fast-evolving area of gambling has brought new challenges to communities, problem gambling treatment providers, and researchers in the field of addictive disorders. It also remains an issue for regulators and policy makers.

## Data Availability Statement

The original contributions presented in the study are included in the article/supplementary materials, further inquiries can be directed to the corresponding author/s.

## Author Contributions

MG-L and KG-M conducted the literature review and wrote the article. DLev, DLe, and J-YL contributed to the method and the drafting, and reviewed the article. All authors contributed to the article and approved the submitted version.

## Conflict of Interest

The authors declare that the research was conducted in the absence of any commercial or financial relationships that could be construed as a potential conflict of interest.
